# Prediction of Protein Structural Features from Sequence Data Based on Shannon Entropy and Kolmogorov Complexity

**DOI:** 10.1371/journal.pone.0119306

**Published:** 2015-04-09

**Authors:** Robert Paul Bywater

**Affiliations:** Magdalen College Oxford OX1 4AU, England; Universidad de Granada, SPAIN

## Abstract

While the genome for a given organism stores the information necessary for the organism to function and flourish it is the proteins that are encoded by the genome that perhaps more than anything else characterize the phenotype for that organism. It is therefore not surprising that one of the many approaches to understanding and predicting protein folding and properties has come from genomics and more specifically from multiple sequence alignments. In this work I explore ways in which data derived from sequence alignment data can be used to investigate in a predictive way three different aspects of protein structure: secondary structures, inter-residue contacts and the dynamics of switching between different states of the protein. In particular the use of Kolmogorov complexity has identified a novel pathway towards achieving these goals.

## Introduction

In order to fulfil their mission proteins have many functions including the need in the first place to fold correctly. “Fold correctly” does not mean or should not mean, as in the parlance of much of the protein research literature, to adopt “the native structure” because that term not only lacks a rigorous and universally agreed definition, but it is always referred to in the singular. I will instead define correctly folded structures as “biologically relevant structures” and there will be for a given protein at least two of these one active and the other inactive or in a resting state [1–3]. In general, the active state (A) will have some bound “small molecule” (SM) ligand and will often therefore have a more “closed” and compact structure while the resting or inactive state (I) will often have a more open structure. Thus two of the principal functions of a protein are the ability to fold into one or other of these two conformational states and to be able to reach one state from the other. Predictions of protein folding must take account of this. The other principal functions are: the need for the protein to get to the correct locus inside or outside the cell, or within the cell membrane (these all require recognition sites on the surface, either for other proteins, for nucleic acids or for lipids—indicating the required destination, much in the manner of a postal “address label” or “barcode”), recognition and binding of SM ligands (substrates, in the case of enzymes, agonists in the case of receptors), catalysis (in the case of enzymes), binding sites for metal ions (critical for many catalytic functions in enzymes and regulatory functions in G-protein coupled receptors, for example), secondary SM binding sites for cofactors or allosteric ligands. Many of these functions depend on postranslational modifications that in turn require some kind of recognition site on the surface for that to happen. Thus any given protein has many functions and these are all encoded in the gene for that protein. The residues along the protein chain are severally responsible for these different protein functions but in a disjoint manner, as a sort of multiplex and aperiodic version of the σκυτάλη secret codes of ancient Sparta. For protein folding itself it has been suggested that only a few key residues are necessary [4–9] and the present work throws light on where these might be located within a carefully selected representative set of protein structures. The focus here is on folding into the said biologically relevant structures and the ability to switch between them under ambient conditions (“ambient” being defined here as: compliant with the metabolic or endocrinological status of the cell and subject to whatever limits are imposed by epigenetic imperatives). The work is conducted at three levels: secondary structure propensities, the three-dimensional structures and switching between these structures. Lastly, the residue-residue contacts necessary to preserve the integrity of the three dimensional structures are considered.

## Measures of Sequence Diversity

### Variability, Shannon entropy and Kolmogorov complexity

Critical to the understanding of this work is the notion of sequence variability at a given residue position in multiple sequence alignments and the corresponding notions of information content, or complexity, at that site. For the latter, two alternative approaches are used here: sequence entropy, defined as the normalised Shannon entropy of the frequency of residue types populating a given position in the primary sequence [10,11] and, introduced here for the first time in protein folding studies, the Kolmogorov complexity [12] of the array of residue types at that position.

Sequence variability and entropy have previously been described and used in several penetrating studies of protein structure and function. The authors of these studies [10,11] distinguish the two with the following statement: “Sequence entropy is a measure of information present in an alignment, whereas sequence variability represents the mutational flexibility at that position”. Another way of putting this is to state: entropy measures what is required at a given site, variability measures what can be tolerated at that site [3]. In seminal papers [10,11], it was shown how variability (VAR) and entropy (ENT) vary in a systematic manner according to location within the protein structure. Sites which have the lowest ENT and VAR tend to be tightly clustered at the active sites of enzymes or the endogenous agonist binding sites of G-protein-coupled receptors, consistent with the notion that these sites do not tolerate introduction of residue types that are not capable of conserving the required function at that site. Other positions in the protein can tolerate a larger influx of diverse residue types depending on how these affect performance of the assigned function for that site, and always, of course, constrained by the evolutionary selection pressures on that function and on the corresponding residue positions.

There are more ways to quantify information than, for example, Shannon entropy. One method that is increasingly coming into use in physics, chemistry and econometrics [12] and bioinformatics [13,14,15] is the notion of Kolmogorov complexity (KOL). In bioinformatics it has mostly been used in the context of systems biology and alignment-free similarity measures [13,14,15] but its use in the field of protein folding, as described herein, is novel. Kolmogorov complexity can be defined [16] as **K(x)**, the most compact compression of **x**, given by **K(x) = min|p|:U(p) = x** where **U** is a universal Turing machine. A simple implementation in practice is **K(x) = min|p|:L(p) = x** where **L** is a suitable compression algorithm such as *bzip2*, a commonly used tool for file compression and which has been used for another purpose elsewhere in the pages of this journal [12]. In the present work, input data was obtained using the PredictProtein program [17] which delivers a multiple sequence alignment in which each row represents one residue position. Each complete row of this multiple alignment spanning over all the orthologs in the alignment was read into a unique file and the file compressed with bzip2 as described in **Methods.** The size of the resulting file is now the desired **K(x)** for that row. Here, the relative size (the ratio **K(x)** of to **x**) was used; this normalisation was employed in order that values at different residue positions could be compared. The resulting KOL complexity scores were used in the subsequent analyses, alongside VAR and ENT.

The first task was to investigate to what extent VAR, ENT and KOL correlate with secondary structure. PredictProtein delivers not only the multiple sequence alignments mentioned above, but secondary structure and other structural information both experimental and predicted by neural networks, but also the VAR and ENT values used throughout this work. KOL was calculated for the entire alignment at each position for each protein using the *bzip2* algorithm as described in **Methods**.

### Location of stretches of secondary structure

Secondary structure prediction algorithms have become ever more sophisticated, with the latest best “scores” still hovering around the 80% level [18]. However, as pointed out earlier [3] It is probably unlikely that this threshold will ever be crossed until neural nets are separately trained on A and I structures, since A and I will in most cases have slightly different patterns of secondary structure. This issue is planned to be addressed in a forthcoming paper, here, a single set of secondary structure values for each pair of proteins is used, as described immediately below and under **Methods**.

Codon bias is widely recognised as playing a role in optimizing the efficiency of translation [19]. Whether or not there is any influence of synonymous codon usage on protein 2D or 3D structure is still an unresolved issue, but in one study [20] it was stated that synonymous codons carry much less structural information in prokaryotes than in eukaryotes. More recent work [21] supports the contention that “slow codons” tend to accumulate at SSE boundaries, although not at domain boundaries. These studies contradict earlier findings [22] where no correlation was found between the positioning of rare codons and the location of SSEs but rather from the similarity of codons coding for very abundant amino acid residues at the N- and C-termini of helices and sheets. There is clearly an issue at stake here which prompted the first of the questions being asked in this paper: are there signals that mark out the beginnings and ends of SSEs? There are two complementary ways, *a priori*, to investigate this: study sequences at the DNA level, which has already been done [19–22], or, as here, investigate how genetic drift has influenced the amino acid sequence patterns that have survived. Here I report studies of to what extent, if any, VAR, ENT and KOL correlate with SSE boundaries.

**Fig 1:**
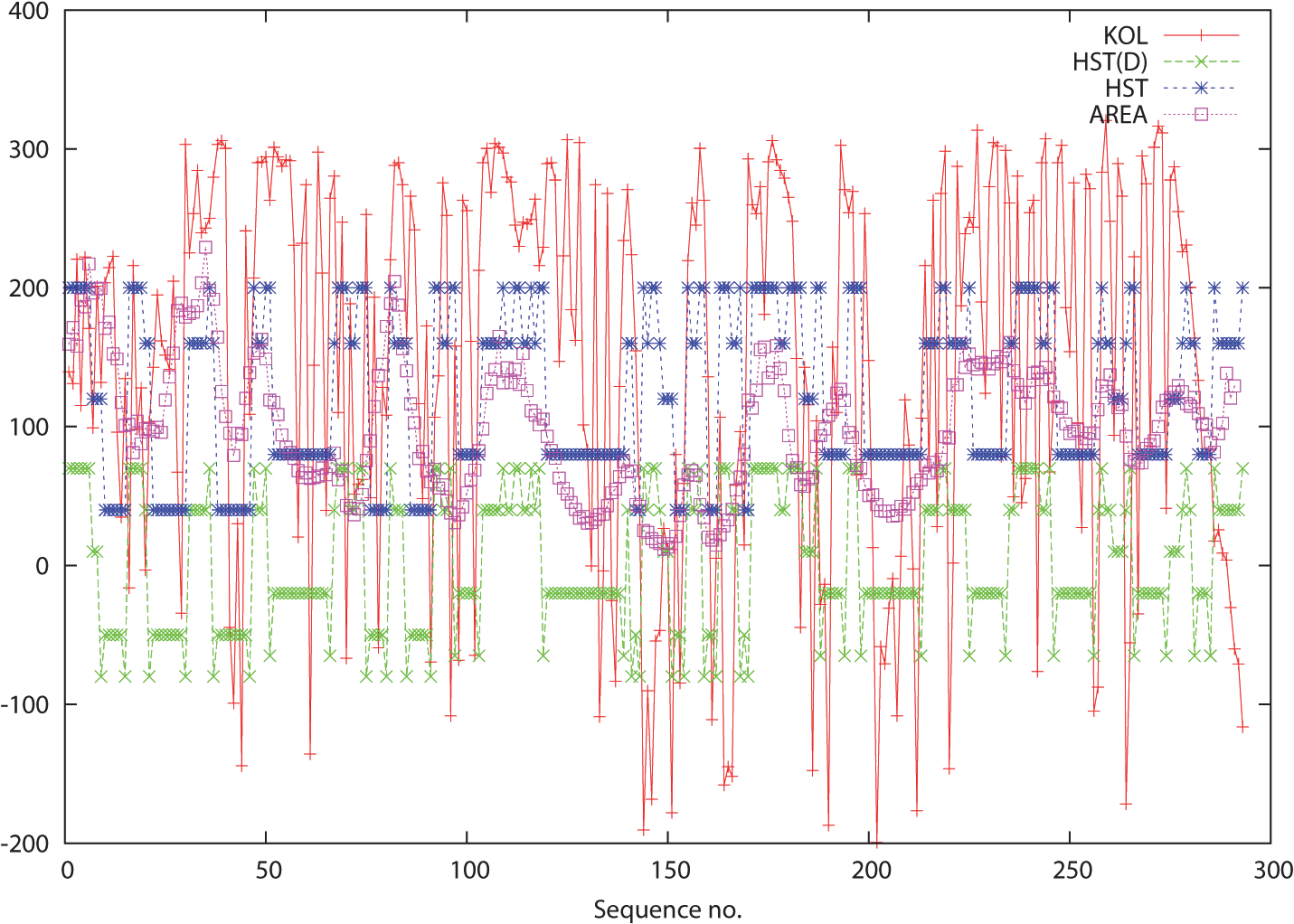
Data for proteins: 2auha and 2b4sb. The abscissa is residue number in the primary sequence and the ordinate is the score for the various parameters KOL, HST(D) and AREA. These are defined in the text and are identified in the key at the top right of each figure.

The results can be seen in Fig 1 (and figures A, D, G, J, M, P, S, V, Y in S1 File) where SSEs are plotted schematically using the HST(C) model [23] against the residue number. The HST(C) assignments were made using the WHAT IF program [24] and for plotting purposes S was assigned a nominal value 1, H = 2, the 3_10_ helix (not a member of the original HST model, but included in the WHAT IF version) = 3, T = 4 and C = 5). As a device for making the “termini of SSEs” easier to identify, the HST data was converted into a new set of data HST(D) (which stands for “HST Differentiated”). This took the form of assigning new values to the beginning and end residues of stretches of S and H: 0 for S and 0.5 for H. In this way the plots now descend below the HST plots at these residues forming easily identifiable “antispikes” (“anti” because lower values are associated with greater conservation). The following general remarks can be made:

- There is a clear preponderance of low-valued KOL (also VAR, ENT, not shown in figures) signals at SSE termini corroborating the need for a high degree of conservation.
- This tendency has been made easier to pick out in Fig 1 and figures A, D, G, J, M, P, S, V, Y in S1 File, where the HST(D) “antispikes” are shown alongside the HST originals.
- Within SSEs, KOL values are usually lower (also VAR, ENT, not shown in figure), likewise meaning greater conservation.
- Turn regions are typically marked out by much higher values for KOL (same for VAR, ENT, not shown in figure), and coil regions are marked by even higher spikes.
- A slight exception to the above can be seen in figures J, K and L in S1 File. The termini are not picked out well, but the SSEs are. This protein is very disordered in the N-terminus (because it binds to DNA, which was not present in the crystal structure) and KOL predicts this disorder (or rather, that there will be disorder if DNA is absent). This is discussed further below.

Although these correlations are clearly visible, there is no simple way to measure this correlation quantitatively since secondary structure can not be expressed in simple scalar terms.

But there is a parameter that can be used to capture the essentials of the variation in backbone geometry along the polypeptide chain. Neighboring CA atoms are always at a constant distance of 3.81 Å from each other but the distances between the i^th^ and (i+2)^th^ CA atoms is not constant but is in an intimate way dependent on the backbone geometry (ϕ and ψ dihedral angles). The influence of secondary structure becomes amplified when a triangle is formed between the i^th^ and (i+2)^th^ CA atoms and the global centre of gravity (CG) of the protein as has been demonstrated earlier [25]. This area acts as a proxy for SSE and has the additional merit that it can be treated as a variable along the polypeptide chain, rather than just a classifier. The triangle areas are plotted against residue number. The algorithm used to make these calculations is given in the Appendix which is provided as supporting information in S1 File. The results are plotted in Fig 1 for the insulin receptor (and figures A, D, G, J, M, P, S, V, Y in S1 File for the other proteins). The areas of these triangles along the protein chain are very sensitive to secondary structure. Again, VAR, ENT and KOL all anticipate the behavior of the triangle areas with high fidelity: small areas (low values along the ordinate) correlate with compact SSEs such as helices, larger areas with turn and coil regions. The area curve follows a “meander” that is in synchrony with the KOL values (VAR and ENT not shown, but the latter two are similar). The corresponding correlation coefficients for VAR, ENT and KOL with the triangle areas (“AREA”) are listed in Table 1. Note: there is a slight rightwards displacement or offset because AREA is calculated for residues i and i+2 for each i (that will tend to make the correlations look “weaker”).

**Table 1:** Estimating secondary structure and other structural parameters.

Pairs of variables for correlation		PDB i.d.s									
		2b4sb & 2auha	1ybib& 1ybia	1ye3 & 1n8ka	1ulka& 1ulkb	1kx5a& 1kx5e	1tpda& 5tim_	1ftja & 1fw0a	1ewka& 1ewkb	1bpxa& 1bpya	1aonn& 1xck
		Number of protein sequences									
		1275	1275	2851	1058	334	1112	2694	2561	692	1940
		SSE frequency									
		31/15 & 32/17	2/50 (9) & 2/47 (9)	24/24 (6) & 24/24 (6)	10/10 (12) & 6/10 (12)	47/0 & 49/0	39/16 & 39/15	35/18 & 34/18	33/19 & 33/19	44/15 & 43/15	48/13 & 50/18
		MW (kDa)									
		33.2	32.8	39.8	13.7	15.3	26.7	28.8	50.6	37.1	55.2
		CATH class									
		3.30.200.20	2.80.10.50	3.40.50.720	3.30.60.10	1.10.20.10	3.20.20.70	3.40.190.10	3.40.50.2300	3.30.210.10	3.30.260.10
		Figure numbers									
		1–3	A,B,C	D,E,F	G,H,I	J,K,L	M,N,O	P,Q,R	S,T,U	V,W,X	Y,Z,Ø
ENT	VAR	0.97	0.96	0.96	0.98	0.96	0.97	0.97	0.95	0.95	0.92
KOL	ENT	0.88	0.25	0.95	0.89	0.93	0.93	0.77	0.68	0.80	0.74
KOL	VAR	0.84	0.24	0.90	0.85	0.91	0.90	0.72	0.62	0.75	0.67
HST	KOL	0.08	0.05	0.11	0.05	0.16	0.03	0.18	0.06	0.08	0.10
HSTD	KOL	0.06	0.03	0.11	0.05	0.13	0.06	0.11	0.04	0.00	0.03
AREA	ENT	0.46	0.15	0.14	0.23	0.33	0.45	0.10	0.10	0.38	0.31
	VAR	0.43	0.13	0.12	0.21	0.29	0.40	0.10	0.09	0.29	0.22
	KOL	0.45	0.06	0.07	0.32	0.22	0.39	0.16	0.08	0.33	0.26
BVLA	ENT	0.26	0.16	0.16	0.08	0.44	0.26	0.31	0.21	0.23	0.32
	VAR	0.22	0.14	0.14	0.06	0.40	0.24	0.31	0.21	0.23	0.29
	KOL	0.24	0.22	0.04	0.24	0.26	0.24	0.21	0.11	0.14	0.42
BVLI	ENT	0.33	0.02	0.22	0.09	0.47	0.26	0.43	0.29	0.28	0.29
	VAR	0.33	0.01	0.17	0.08	0.42	0.26	0.44	0.28	0.24	0.27
	KOL	0.35	0.12	0.20	0.25	0.29	0.25	0.38	0.01	0.16	0.39
OACA	ENT	0.57	0.21	0.29	0.34	0.01	0.36	0.15	0.35	0.38	0.37
	VAR	0.55	0.16	0.27	0.34	0.23	0.35	0.16	0.34	0.38	0.32
	KOL	0.49	0.05	0.24	0.18	0.07	0.33	0.09	0.11	0.28	0.27
OACI	ENT	0.58	0.21	0.28	0.35	0.07	0.34	0.18	0.37	0.35	0.33
	VAR	0.56	0.16	0.27	0.35	0.09	0.33	0.20	0.35	0.37	0.28
	KOL	0.53	0.06	0.24	0.17	0.10	0.30	0.10	0.13	0.24	0.22
DISP	ENT	0.05	0.18	0.15	0.03	0.26	0.15	0.28	0.12	0.04	0.27
	VAR	0.05	0.14	0.13	0.04	0.25	0.12	0.31	0.12	0.03	0.24
	KOL	0.09	0.03	0.19	0.02	0.16	0.17	0.12	0.22	0.02	0.41
BVLC	ENT	0.15	0.17	0.15	0.02	0.17	0.08	0.36	0.02	0.19	0.20
	VAR	0.12	0.15	0.12	0.02	0.18	0.05	0.37	0.03	0.15	0.20
	KOL	0.13	0.32	0.22	0.01	0.01	0.06	0.36	0.22	0.10	0.28

Below the PDB I.d.s of the protein pairs studied are in order: the number of sequences in each alignment (as treated by the PredictProtein program), the relative frequency of secondary structures (%alpha-helix/%beta-strand) in each member of the protein pair (Note: for 1ulka/1ulkb, a 3_10_-rich protein, and likewise 1ybib/1ybia and 1ye3/1n8ka the 3_10_ content is added in parentheses), the molecular weight of the protein, the CATH class (SCOP classes are not as useful, since the SSE data already alludes to this kind of classification) and a key to the numbers of the corresponding figures. The correlation data for each protein (pair) for each type of analysis completes the table. For HST/HSTD, only data for KOL are shown.

The conclusion from these two studies is that VAR, ENT and KOL all correlate with SSE patterns and backbone geometry which suggests ways of using them in a predictive fashion for secondary structures. The use of KOL, in particular, at the DNA level would clearly be of interest and this work has already been commenced.

### Prediction of three-dimensional structures

**Fig 2:**
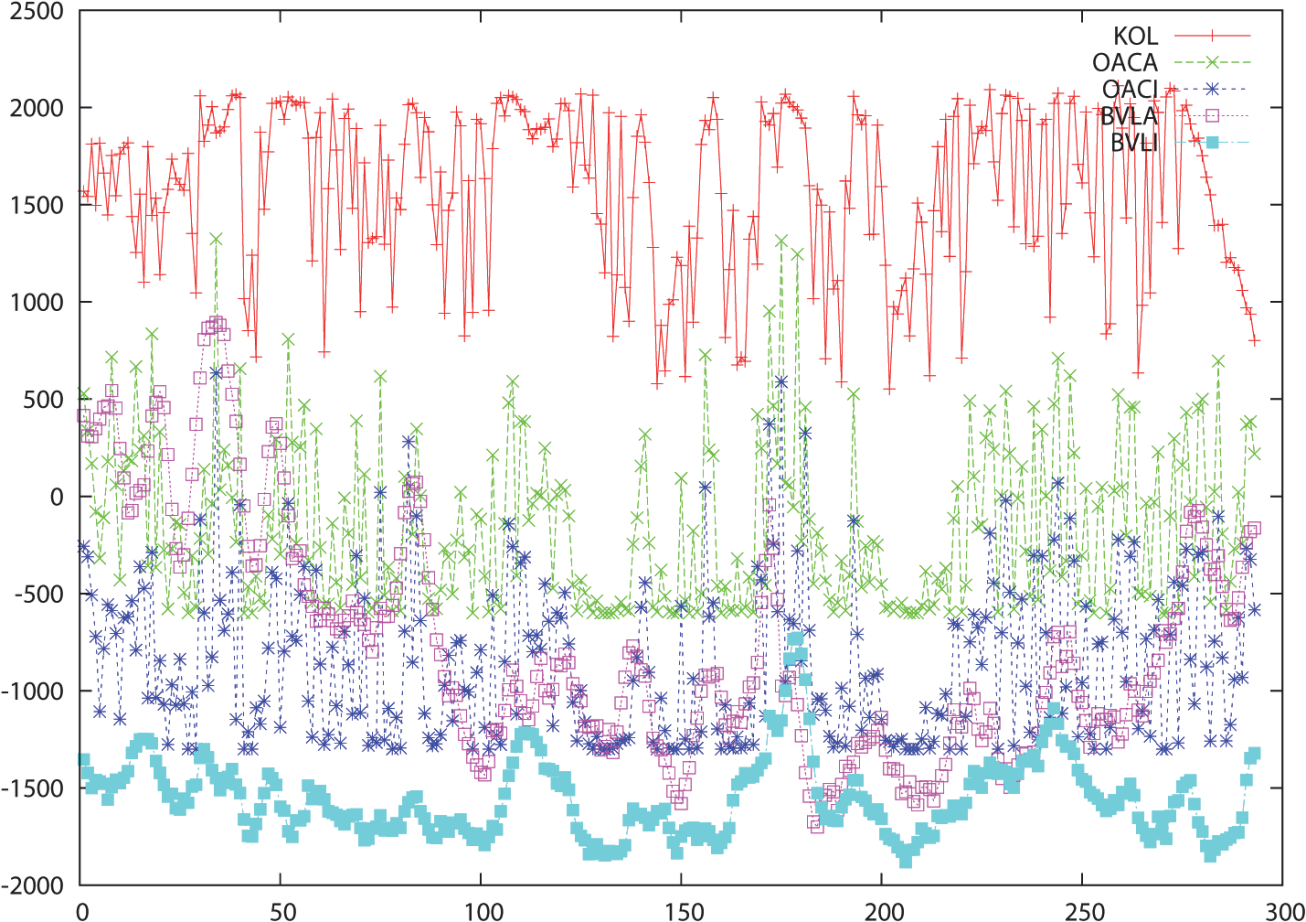
Data for proteins: 2auha and 2b4sb. The abscissa is residue number in the primary sequence and the ordinate is the score for the various parameters KOL, OACA, OACI, BVLA and BVLI. These are defined in the text and are identified in the key at the top right of each figure.

Moving on to considerations of three dimensional structure, a similar behavior is observed for KOL (similarly for VAR and ENT, but not shown graphically) in synchrony with solvent accessibilities (calculated from crystal structure coordinates using WHAT IF) and B-values (experimental) for these proteins. These (OACA/OACI and BVLA/BVLI respectively) are plotted separately for the A and I structures in Fig 2 for the insulin receptor (and figures B, E, H, K, N, Q, T, W, Z in S1 File for the other proteins) with OACA/OACI denoting the accessibilities for A and I respectively and BVLA/BVLI likewise for the B-values. Higher B-values occur, not unexpectedly, when both variability and entropy are higher but the same is true for accessibilities, reflecting the notion that surface residues are more mobile with fewer intramolecular contacts and thus more readily mutated than those in the well-packed core. How far such correlations can be used for structure prediction remains to be investigated but together with the contact predictions discussed below there is every reason to include this data in prediction work. The members of the pairs OACA/OACI and BVLA/BVLI both correlate with KOL but slightly differently. Again, this calls for deeper investigation.

**Fig 3:**
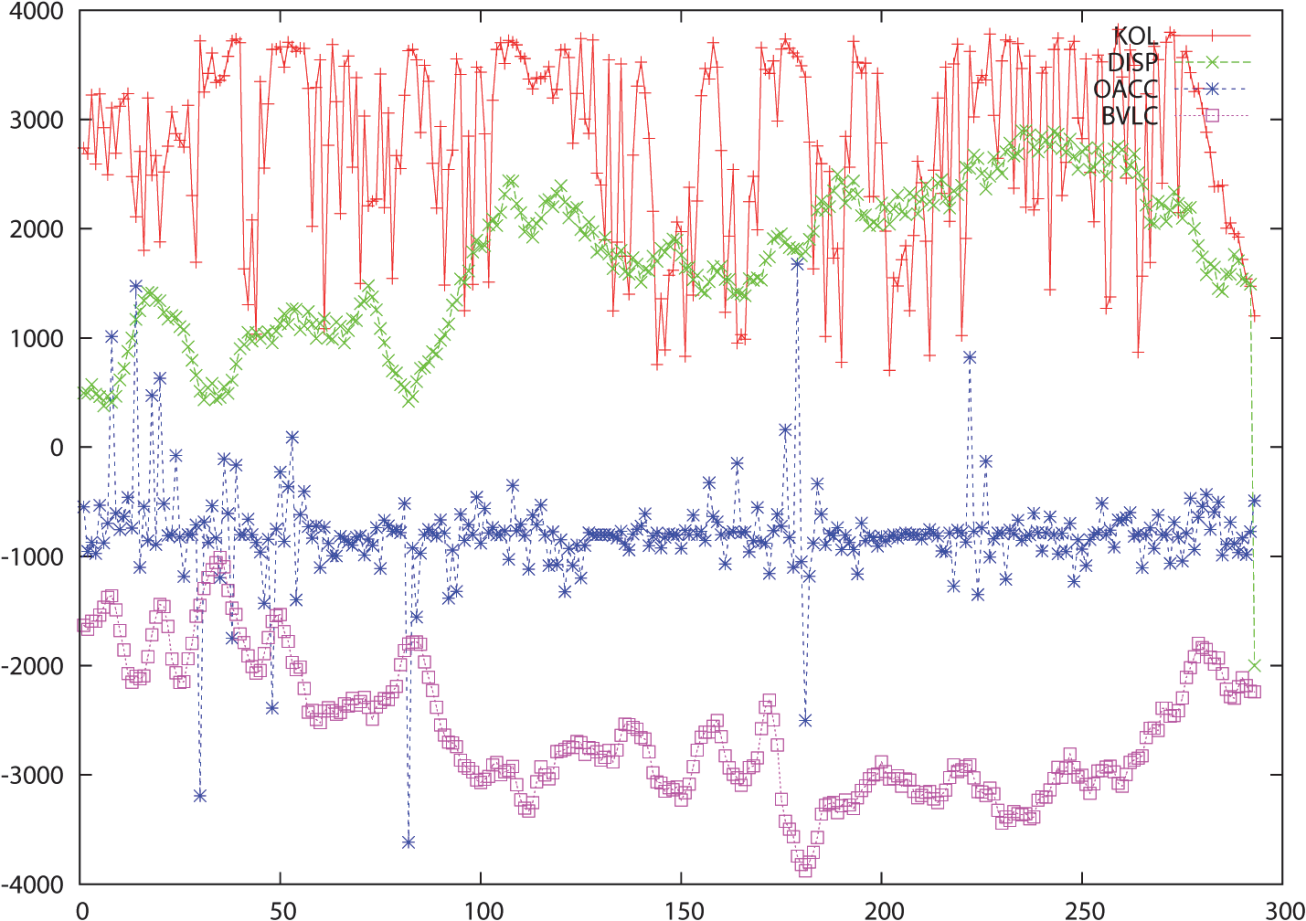
Data for proteins: 2auha and 2b4sb. The abscissa is residue number in the primary sequence and the ordinate is the score for the various parameters KOL, DISP, OACC and BVLC These are defined in the text and are identified in the key at the top right of each figure.

The previous figures gave clear indications that there were differences in the way that the A and I structures are programmed by the genetic sequence so it seemed relevant to ask not only to what extent there was any correlation between accessibilities and B-values for these structures but to what extent the difference itself was influenced by the genomic data. When the differences are considered, there is a clear correlation not only for accessibility differences (OACC = abs(OACA—OACI)) and B-values (BVLC = abs(BVLA—BVLI)), but also the relative atomic displacements in Å between cognate CA atoms in the A and I structures (DISP). These are shown in Fig 3 for the insulin receptor (and figures C, F, I, L, O, R, U, X, Ø in S1 File for the other proteins). The fact that there is a correlation with these differences explains why attempts to determine linear correlations between VAR, ENT and KOL and the various protein structure parameters did not always give a clear-cut picture (Table 1). There is clearly (from the graphics) a significant correlation, but since we are dealing with a multivariate system, the individual pairwise correlations will necessarily be lower. It is important to note that the DISP values are differences between 3D coordinates for two cognate structures so that their correlations with VAR, ENT or KOL can just as well be, and sometimes are, anticorrelations. The criterion by which these comparisons are to be judged is the tempo, to use a musical analogy, of the changes along the sequence axis and not the sense of the displacement (the absolute value, in other words).

**Fig 4:**
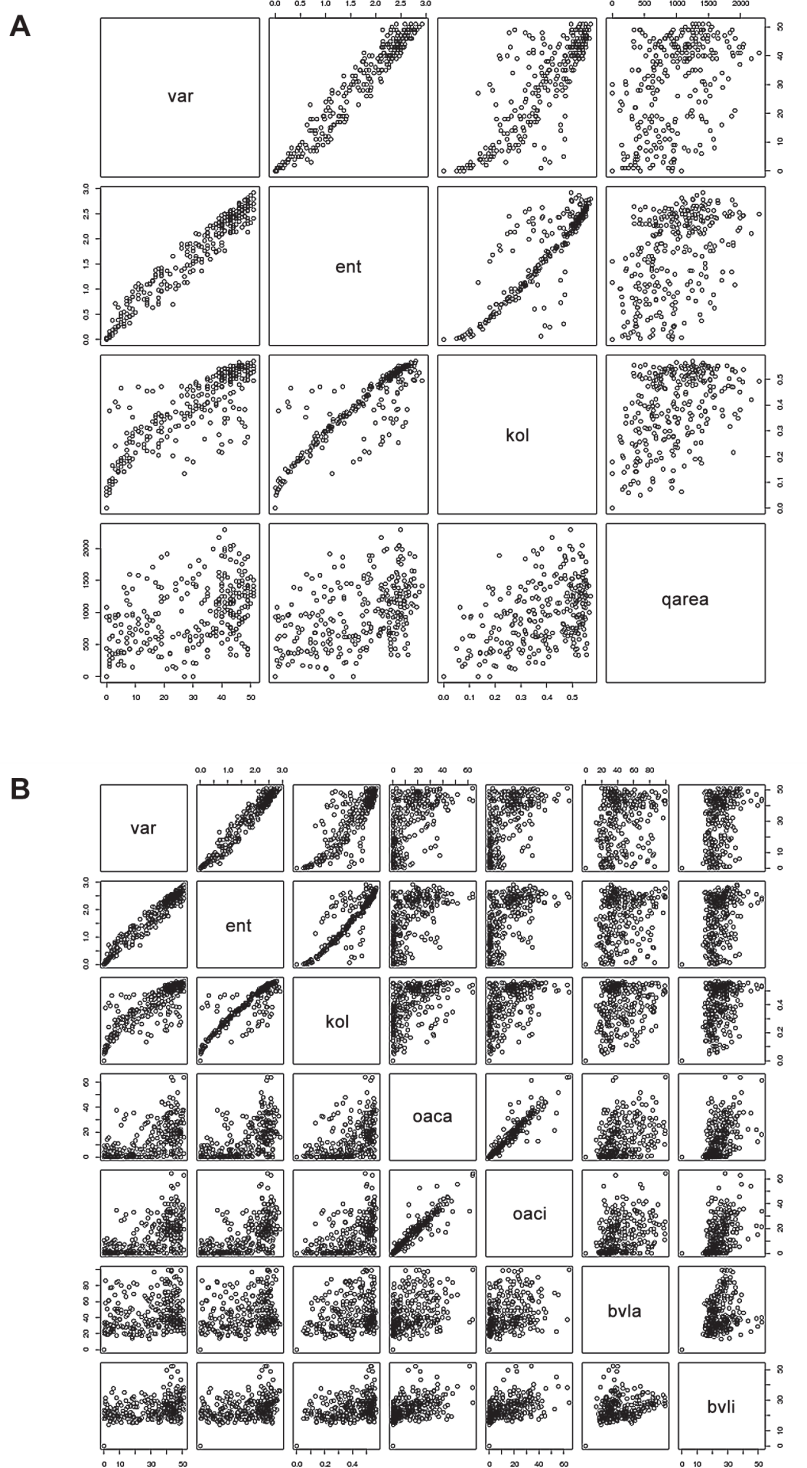
The correlation diagrams from the R program showing correlations between the variables for the protein pair 2auha and 2b4sb. Corresponding figures for the other members of the set are available from the author. Fig 4A shows correlations between VAR, ENT, KOL and AREA (labeled qarea in the diagram). Fig 4B shows correlations between VAR, ENT, KOL and other variables as labeled.

The correlation data for the two preceding studies is to be found graphically in Fig 4 (for the protein pair 2auha and 2b4sb) and in tabular form for all protein pairs in Table 1.

The final set of studies was more focused on 3D fold prediction as such. For the purposes of this work, in keeping with established practise, as in earlier published studies using the correlated mutation approach [26–32] it was considered adequate to calculate 2-dimensional contact maps in which the predictions provided by VAR/ENT or KOL can be compared with the experimentally determined map of residue pair contacts. In principle, the 3-dimensional structure of the protein can always be reconstructed from such contact maps using distance geometry [33–35] and in this case there is considerable extra information (predictions of secondary structure and accessibility in particular) which would add additional confidence to the results of attempts to compute the 3D structure (a planned future extension of this work). For comparison purposes, the contact maps are anyway of considerable value because they are invariant with respect to how the 3D coordinates are embedded in 3D space. The calculations for VAR and ENT were performed by restricting the VAR, ENT values so that they lie within the “1:2 box” identified [10,11] as the region that most strongly corresponds to fold preservation residues, i.e the residues in the subset of the disjoint set of all residues referred to above. The filter that was applied restricted VAR to between 0.5 and 1.5 and ENT to between 50 and 60. For the KOL case, KOL was plotted against itself and a (default values) filter of 1.5 (lower bound) and 3.5 (upper bound) was applied.

**Fig 5:**
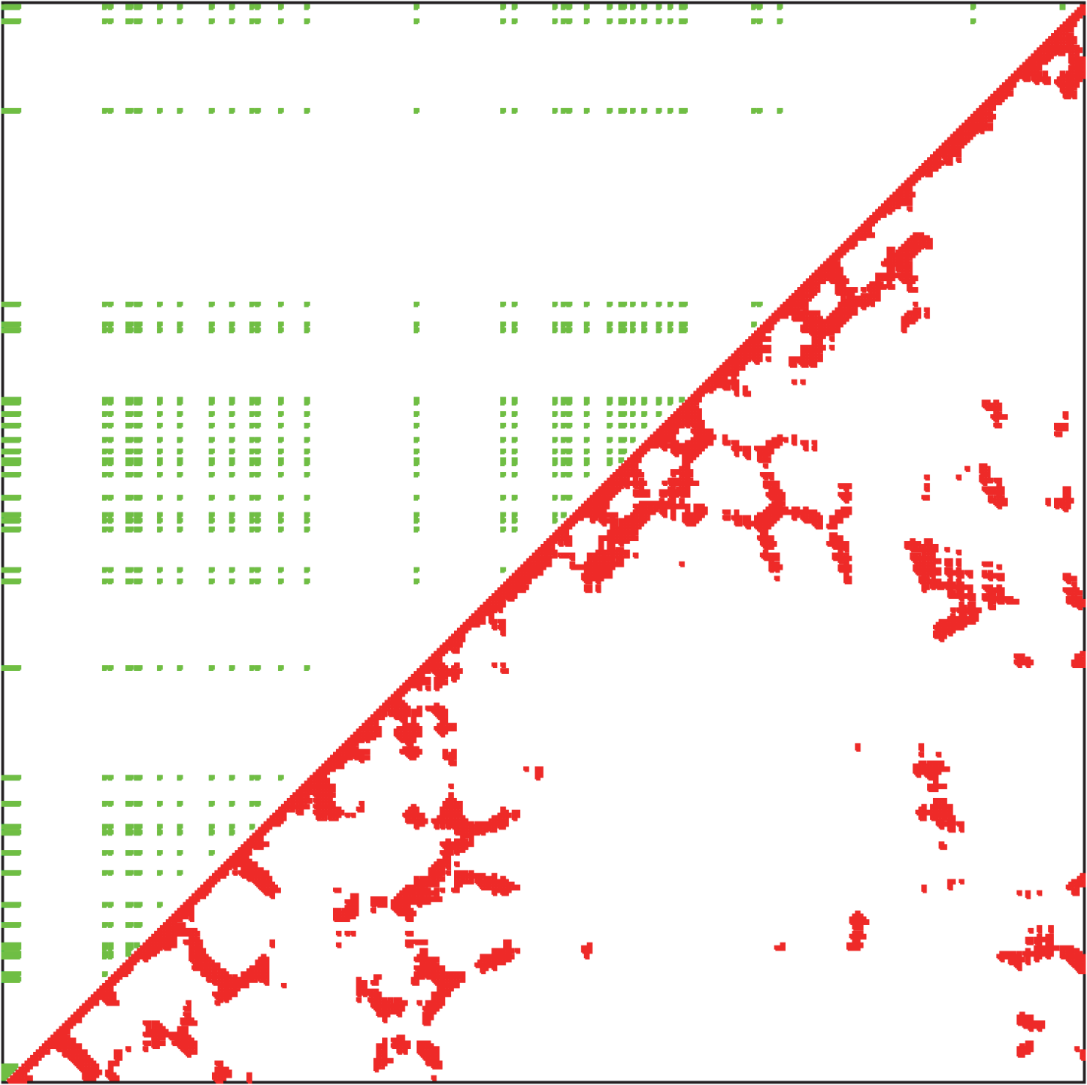
Predicted and experimental contact maps shown for only one pair: 2auha and 2b4sb.

The results are shown in graphical form for one single case only (for the insulin receptor, Fig 5) as an example, since the data is more completely and rigorously represented quantitatively, in tabular form (Table 2), where the number of true positives (TP), false positives (FP), false negatives (FN) and true negatives (TN) are given for all 20 proteins, together with the corresponding Matthew's Correlation Coefficients (MCC) defined [36,37] as

MCC=(TP*TN–FP*FN)/√((TP+FP)*(TP+FN)*(TN+FP)*(TN+FN))

**Table 2:** Statistics for prediction of 3D contacts.

Protein	Prediction method	TP	FP	FN	TN	MCC
2auha	VRN	122	1294	714	40648	0.0890
	KLM	269	1264	506	40739	0.2275
2b4sb	VRN	124	1283	724	40647	0.0904
	KLM	270	1253	516	40739	0.2273
1ybia	VRN	90	1274	81	38741	0.1778
	KLM	361	1301	844	37680	0.2280
1ybib	VRN	88	1273	80	38745	0.1755
	KLM	362	1299	845	37680	0.2286
1n8ka	VRN	482	1880	5631	61758	0.0771
	KLM	589	1880	5622	61660	0.1006
1ye3	VRN	494	1905	5607	61745	0.0791
	KLM	601	1905	5598	61647	0.1024
1ulka	VRN	54	573	273	6975	0.0658
	KLM	91	586	261	6937	0.1332
1ulkb	VRN	58	562	262	6993	0.0783
	KLM	91	574	249	6961	0.1400
1kx5a	VRN	276	343	1904	6522	0.1298
	KLM	96	360	343	8246	0.1737
1kx5e	VRN	274	348	1898	6525	0.1275
	KLM	95	365	337	8248	0.1723
1tpda	VRN	529	1086	5953	23308	0.0678
	KLM	379	1043	2526	26928	0.1298
5tim_	VRN	543	1111	5928	23294	0.0694
	KLM	390	1068	2501	26917	0.1329
1ftja	VRN	293	1094	1854	29912	0.1244
	KLM	262	1102	1043	30746	0.1627
1fw0a	VRN	290	1084	1864	29915	0.1232
	KLM	267	1092	1053	30741	0.1656
1ewka	VRN	312	2110	3348	94806	0.0775
	KLM	523	2110	5270	92673	0.0993
1ewkb	VRN	306	2081	3377	94812	0.0760
	KLM	528	2081	5299	92668	0.1009
1bpxa	VRN	335	1337	4282	47021	0.0725
	KLM	159	1395	1020	50401	0.0943
1bpya	VRN	333	1329	4291	47022	0.0721
	KLM	159	1386	1028	50402	0.0943
1aonn	VRN	97	2534	627	133768	0.0610
	KLM	390	2491	1250	132895	0.1663
1xck	VRN	94	2570	663	133699	0.0565
	KLM	392	2526	1214	132894	0.1681

TP, FP—true and false positives respectively, FN,TN—corresponding negatives, MCC—Matthews Correlation Coefficient.

What is most noticeable is that KOL performs much better than VAR/ENT for every single protein on the list. The pattern displayed by the “contacts” predicted by KOL seem to reflect the experimental structure better than VAR/ENT, for example, an array of hits long the top horizontal axis that are completely absent in VAR/ENT. In particular, for 3D prediction, KOL seems to be emerging as the method of choice.

### Concluding remarks

It is evident just from a perusal of the data presented here that VAR, ENT and particularly KOL reveal essential features related to protein structure, function and dynamics. In particular, these are the beginnings and ends of SSEs, the SSEs themselves, key sites for dynamical switching between states of the protein and all the others that need to be identified and partitioned from the underlying sequence data. VAR, ENT and KOL are derived only from genomic data yet they anticipate so many of these protein properties. One has now a firm basis for proceeding from anticipation to prediction. The method used here is novel, and it reveals more than just a route to “protein folding”, given that most proteins fold into more than one structure [1–3]. The duality of these folding pathways is revealed by KOL (as well as VAR and ENT).

There are many cases where proteins are partly or even mostly “unstructured” [38], but KOL can even deal with this. For example the apparently odd behaviour of the first 40 residues of the histone proteins (shown in S1 File figures J, K and L) is unerringly predicted by Kolmogorov and can be explained when one understands that this seemingly”unstructured” part of the protein is involved in establishing contacts with DNA in the chromosome, but DNA is absent in the crystal. So, even “unstructure” can be predicted.

Concerning the choice of method, VAR and ENT or KOL: the latter is clearly superior for 3D prediction (contact map data as summarized in Table 2). The statistics for the other structure parameters vary somewhat, in certain cases KOL is superior to VAR/ENT and in others the converse is true (Table 1). It may a bit misleading to stare too closely at these correlations, since it is not certain that these correlations are truly linear. Inspection of the correlation diagrams **(** Fig 4 **)** shows that the KOL data is much less noisy in all cases, with fewer outliers than VAR and ENT. This may indicate a greater inherent fidelity in the KOL data, regardless of what the correlation value is.

Regarding the question of “correlation value”, it is of course apparent from Table 1 that correlation coefficients appear to be on the “low” side, compared to certain current state-of-the-art accomplishments in this area [39]. But it is important to be aware that correlation coefficients in multivariate systems such as this will never come up to the levels that are experienced in statistical computations of correlations between individual pairs of variables as in [39]. Neither VAR, ENT nor KOL can be described as “individual” in this sense. The whole point is that they need, and are beginning to be, partitioned so that their relationship to the different structural parameters that we are interested in can be established. VAR, ENT nor KOL are all derived from purely evolutionary information and evolution has, by its own definition, the task of catering for all of these “structural parameters”. The challenge is now to unravel this information (“partition”, to use the correct mathematical verb) so that we can start to predict the individual parameters from genome data alone. It amounts to a cryptographic problem—see the Appendix provided as supporting information.

A recent review [19] stated that advances toward integrating genomic and proteomic information are essential. It has been the intention in this paper to attempt to make advances of this kind and progress in this direction has been made, as clearly demonstrated in the figures. There is a wealth of information in the data presented here that can be further exploited in the development of prediction algorithms, and the method can be applied to essentially any protein family where accurate multiple sequence alignments of sufficient size are available. Work is underway in several such families including G-protein coupled receptors and the cytochrome P450 family. Preliminary results indicate that these large proteins perform better than smaller proteins under this type of analysis. This is in contrast to all other known protein folding methods where small proteins can be handled but large ones cannot. It is anticipated that the “anticipations” that are revealed by this data will lead to convincing and useful predictions. Recent publications have alluded to an increasingly widely held opinion that the protein folding problem is more or less “solved” [31,32]. There is some truth in this, but not enough. To begin with, the issue of “more than one structure per protein” has not been adequately addressed until now (this paper), although it has been alluded to briefly [32] and the basics are well documented in the DynDom database [1,3]. Methods which improve as larger protein domains are studied are always going to be useful. Finally, new insights are often gained when new methodology is introduced and applied successfully.

## Methods

A basic requirement throughout all work of this kind is a nonredundant set of high resolution crystal structures. A particular requirement for this work was the existence of pairs of crystal structures with conformations that are related by the kind of switch mechanism mentioned above and defined for example by the Dyndom program [1] which determines domain boundaries and hinge regions (http://fizz.cmp.uea.ac.uk/dyndom/). Such a set, having high resolution (R < 2.0 Å) and low B-values (< 50.0) and with sequence identity between the pairs 95% or greater was extracted from the Dyndom database from an earlier and rather different study [3]. This consisted of 20 pairs fulfilling the above criteria. A further restriction was applied for this work, namely 100% sequence identity between the members of the pair. The pairs of proteins used were (four-letter PDB I.d. With appended chain identifier): 2auha/2b4sb, 1ybia/1ybib, 1ye3a/1n8ka, 1ulka/1ulkb, 1kx5e/1kx5a, 5tim_/1tpda, 1ftja/1fw0a, 1ewka/1ewkb, 1bpya/1bpxa and 1xckn/1aong. Data for the first pair (insulin receptor) is shown in the printed version of the paper and the remainder are to be found in the supporting information S1 File.

The WHAT IF program [24] was used for protein modeling and for determination of secondary structure (HST), accessibilities and surface areas. In this paper, HST values for only one of the members of each pair of proteins was used in order to avoid cluttering the figures. (The two sets of HST values are in any case fairly close, although they are distinct, and a special study of these pairwise differences is planned for separate publication.)

The PredictProtein program [17] was employed for producing multiple sequence alignments and predictions of secondary structure, surface accessibility and crystallographic B-values. Furthermore, VAR and ENT data are produced by this program, they can be easily extracted from the plain text version of the output. This was done using a collection of awk, linux shellscripts and fortran routines written for the purpose. Obtaining the KOL data is a little trickier. In order to cater for many thousands of orthologs in a given alignment PredictProtein breaks these into 70-wide blocks of aligned sequences with sequence number, VAR and ENT and other data supplied in vertical columns (i.e. along the “sequence axis”). In order to compute KOL, a complete set of aligned residue data is need at each position in the sequence i.e. for all orthologs so that complete set of alignment data for each position could be restored. This makes it necessary to unwrap the alignment for each position which has to be done semi-manually (a combination of awkscripts and “cut & paste”). The alignment at each residue position was then written to a unique file which was compressed using *bzip2*, thereby providing the compressibility score (Kolmogorov complexity) as defined above for each residue position.

The **R** statistics package [40] was used for statistical calculations and for generating Fig 4.

## Supporting Information

S1 File This file includes figures A, B, and C (Proteins: 1ybia and 1ybib), D, E, and F (Proteins: 1ye3a and 1n8ka), G, H and I (1ulka and 1ulkb), J, K and L (1kx5e and 1kx5a), M, N and O (5tim_ and 1tpda), P, Q and R (1ftja and 1fw0a), S, T and U (1ewka and 1ewkb), V, W, X (1bpya and 1bpxa), Y, Z and Ø (1xckn and 1aong). The following are plotted (KOL always in red). Figures A, D, G, J, M, P, S, V, Y: KOL, HST(D) and AREA. Figures B, E, H, K, N, Q, T, W, Z: KOL, OACA, OACI, BVLA and BVLI. Figures C, F, I, L, O, R, U, X, Ø: KOL, DISP, OACC and BVLC.
(GZ) Click here for additional data file.
