# Supplementary figures and images for: Prediction of Protein Structural Features from Sequence Data Based on Shannon Entropy and Kolmogorov Complexity
Source: PLoS One. 2015 Apr 9;10(4):e0119306. doi: 10.1371/journal.pone.0119306 (PMC4391790; doi:10.1371/journal.pone.0119306)

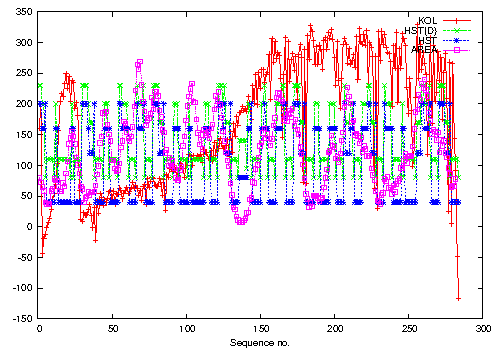

Supplement: S1 File — The following are plotted (KOL always in red). Figures A, D, G, J, M, P, S, V, Y: KOL, HST(D) and AREA. Figures B, E, H, K, N, Q, T, W, Z: KOL, OACA, OACI, BVLA and BVLI. Figures C, F, I, L, O, R, U, X, Ø: KOL, DISP, OACC and BVLC. (GZ) [file pone.0119306.s001.gz › AllFigs/Figure_A.tif]

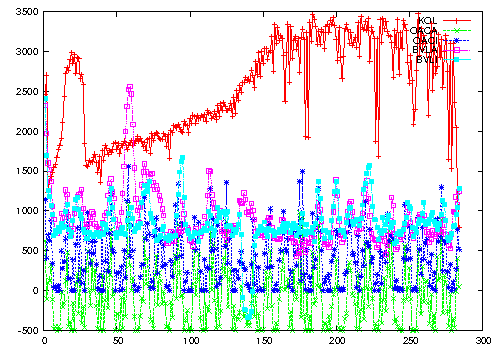

Supplement: S1 File — The following are plotted (KOL always in red). Figures A, D, G, J, M, P, S, V, Y: KOL, HST(D) and AREA. Figures B, E, H, K, N, Q, T, W, Z: KOL, OACA, OACI, BVLA and BVLI. Figures C, F, I, L, O, R, U, X, Ø: KOL, DISP, OACC and BVLC. (GZ) [file pone.0119306.s001.gz › AllFigs/Figure_B.tif]

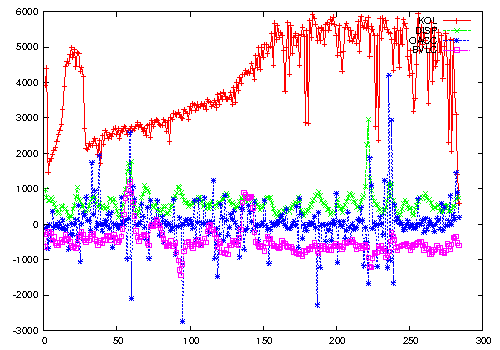

Supplement: S1 File — The following are plotted (KOL always in red). Figures A, D, G, J, M, P, S, V, Y: KOL, HST(D) and AREA. Figures B, E, H, K, N, Q, T, W, Z: KOL, OACA, OACI, BVLA and BVLI. Figures C, F, I, L, O, R, U, X, Ø: KOL, DISP, OACC and BVLC. (GZ) [file pone.0119306.s001.gz › AllFigs/Figure_C.tif]

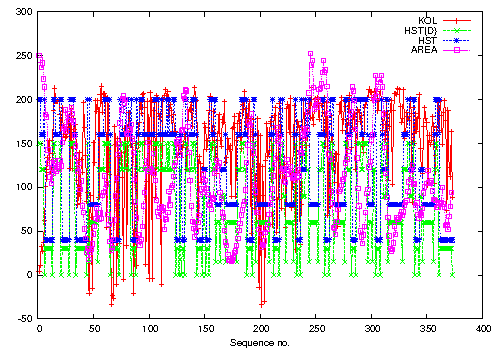

Supplement: S1 File — The following are plotted (KOL always in red). Figures A, D, G, J, M, P, S, V, Y: KOL, HST(D) and AREA. Figures B, E, H, K, N, Q, T, W, Z: KOL, OACA, OACI, BVLA and BVLI. Figures C, F, I, L, O, R, U, X, Ø: KOL, DISP, OACC and BVLC. (GZ) [file pone.0119306.s001.gz › AllFigs/Figure_D.tif]

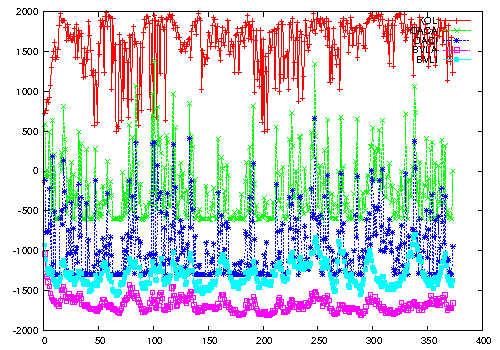

Supplement: S1 File — The following are plotted (KOL always in red). Figures A, D, G, J, M, P, S, V, Y: KOL, HST(D) and AREA. Figures B, E, H, K, N, Q, T, W, Z: KOL, OACA, OACI, BVLA and BVLI. Figures C, F, I, L, O, R, U, X, Ø: KOL, DISP, OACC and BVLC. (GZ) [file pone.0119306.s001.gz › AllFigs/Figure_E.tif]

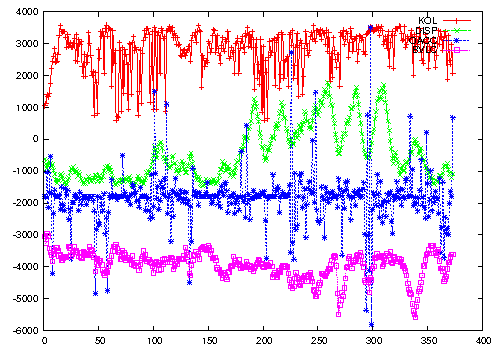

Supplement: S1 File — The following are plotted (KOL always in red). Figures A, D, G, J, M, P, S, V, Y: KOL, HST(D) and AREA. Figures B, E, H, K, N, Q, T, W, Z: KOL, OACA, OACI, BVLA and BVLI. Figures C, F, I, L, O, R, U, X, Ø: KOL, DISP, OACC and BVLC. (GZ) [file pone.0119306.s001.gz › AllFigs/Figure_F.tif]

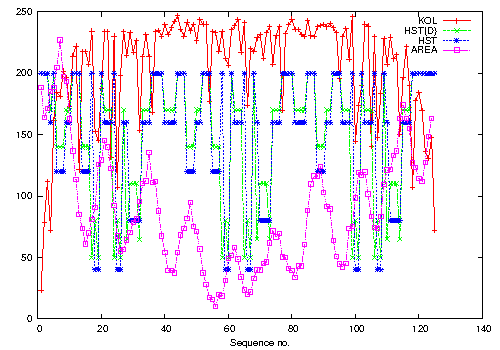

Supplement: S1 File — The following are plotted (KOL always in red). Figures A, D, G, J, M, P, S, V, Y: KOL, HST(D) and AREA. Figures B, E, H, K, N, Q, T, W, Z: KOL, OACA, OACI, BVLA and BVLI. Figures C, F, I, L, O, R, U, X, Ø: KOL, DISP, OACC and BVLC. (GZ) [file pone.0119306.s001.gz › AllFigs/Figure_G.tif]

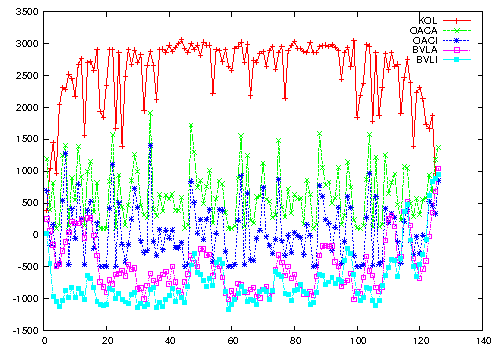

Supplement: S1 File — The following are plotted (KOL always in red). Figures A, D, G, J, M, P, S, V, Y: KOL, HST(D) and AREA. Figures B, E, H, K, N, Q, T, W, Z: KOL, OACA, OACI, BVLA and BVLI. Figures C, F, I, L, O, R, U, X, Ø: KOL, DISP, OACC and BVLC. (GZ) [file pone.0119306.s001.gz › AllFigs/Figure_H.tif]

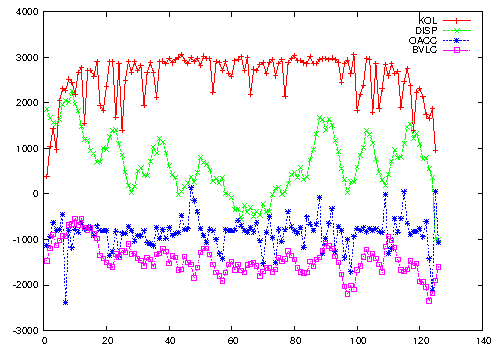

Supplement: S1 File — The following are plotted (KOL always in red). Figures A, D, G, J, M, P, S, V, Y: KOL, HST(D) and AREA. Figures B, E, H, K, N, Q, T, W, Z: KOL, OACA, OACI, BVLA and BVLI. Figures C, F, I, L, O, R, U, X, Ø: KOL, DISP, OACC and BVLC. (GZ) [file pone.0119306.s001.gz › AllFigs/Figure_I.tif]

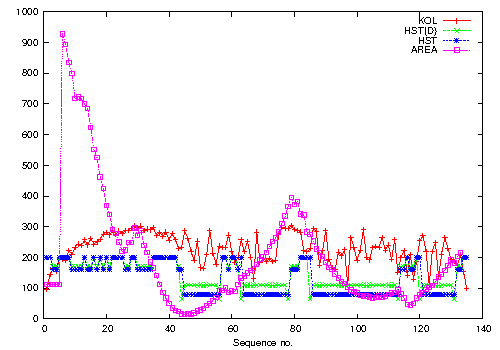

Supplement: S1 File — The following are plotted (KOL always in red). Figures A, D, G, J, M, P, S, V, Y: KOL, HST(D) and AREA. Figures B, E, H, K, N, Q, T, W, Z: KOL, OACA, OACI, BVLA and BVLI. Figures C, F, I, L, O, R, U, X, Ø: KOL, DISP, OACC and BVLC. (GZ) [file pone.0119306.s001.gz › AllFigs/Figure_J.tif]

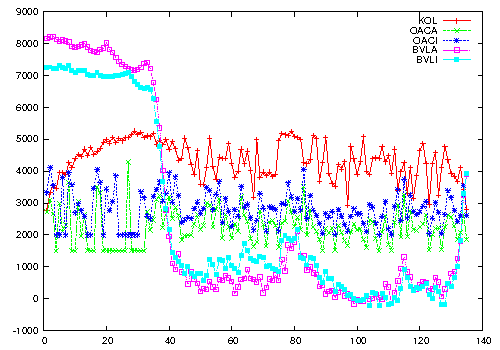

Supplement: S1 File — The following are plotted (KOL always in red). Figures A, D, G, J, M, P, S, V, Y: KOL, HST(D) and AREA. Figures B, E, H, K, N, Q, T, W, Z: KOL, OACA, OACI, BVLA and BVLI. Figures C, F, I, L, O, R, U, X, Ø: KOL, DISP, OACC and BVLC. (GZ) [file pone.0119306.s001.gz › AllFigs/Figure_K.tif]

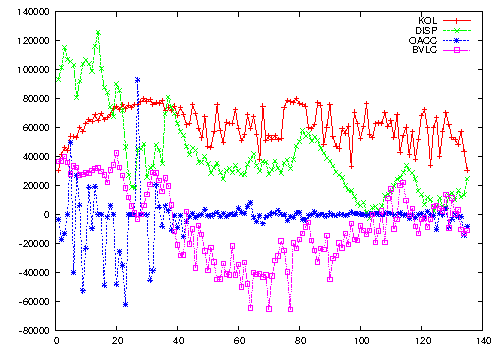

Supplement: S1 File — The following are plotted (KOL always in red). Figures A, D, G, J, M, P, S, V, Y: KOL, HST(D) and AREA. Figures B, E, H, K, N, Q, T, W, Z: KOL, OACA, OACI, BVLA and BVLI. Figures C, F, I, L, O, R, U, X, Ø: KOL, DISP, OACC and BVLC. (GZ) [file pone.0119306.s001.gz › AllFigs/Figure_L.tif]

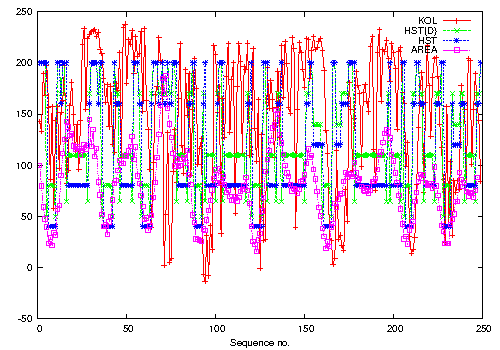

Supplement: S1 File — The following are plotted (KOL always in red). Figures A, D, G, J, M, P, S, V, Y: KOL, HST(D) and AREA. Figures B, E, H, K, N, Q, T, W, Z: KOL, OACA, OACI, BVLA and BVLI. Figures C, F, I, L, O, R, U, X, Ø: KOL, DISP, OACC and BVLC. (GZ) [file pone.0119306.s001.gz › AllFigs/Figure_M.tif]

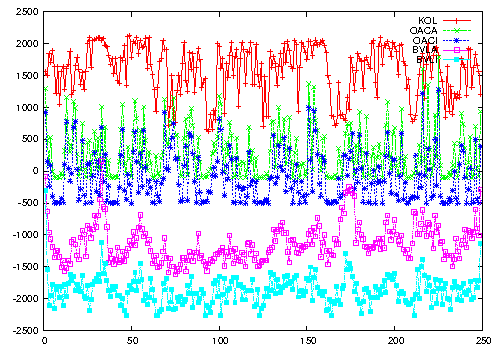

Supplement: S1 File — The following are plotted (KOL always in red). Figures A, D, G, J, M, P, S, V, Y: KOL, HST(D) and AREA. Figures B, E, H, K, N, Q, T, W, Z: KOL, OACA, OACI, BVLA and BVLI. Figures C, F, I, L, O, R, U, X, Ø: KOL, DISP, OACC and BVLC. (GZ) [file pone.0119306.s001.gz › AllFigs/Figure_N.tif]

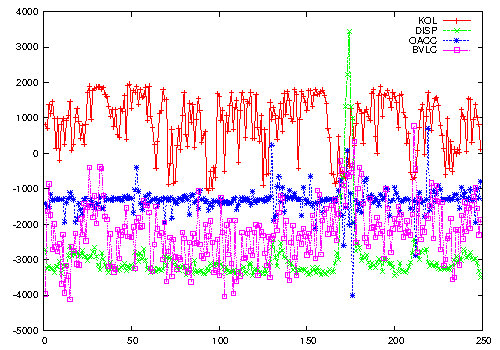

Supplement: S1 File — The following are plotted (KOL always in red). Figures A, D, G, J, M, P, S, V, Y: KOL, HST(D) and AREA. Figures B, E, H, K, N, Q, T, W, Z: KOL, OACA, OACI, BVLA and BVLI. Figures C, F, I, L, O, R, U, X, Ø: KOL, DISP, OACC and BVLC. (GZ) [file pone.0119306.s001.gz › AllFigs/Figure_O.tif]

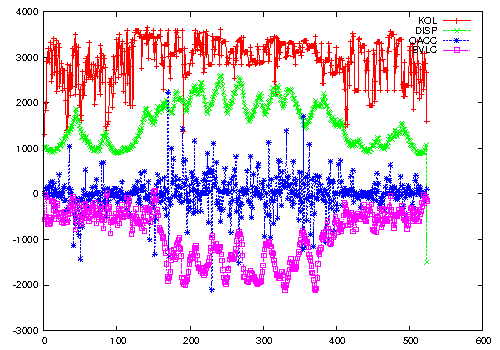

Supplement: S1 File — The following are plotted (KOL always in red). Figures A, D, G, J, M, P, S, V, Y: KOL, HST(D) and AREA. Figures B, E, H, K, N, Q, T, W, Z: KOL, OACA, OACI, BVLA and BVLI. Figures C, F, I, L, O, R, U, X, Ø: KOL, DISP, OACC and BVLC. (GZ) [file pone.0119306.s001.gz › AllFigs/Figure_Ø.tif]

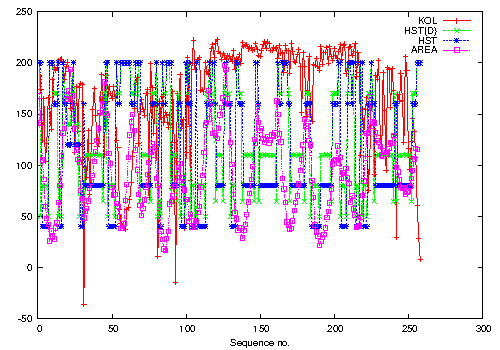

Supplement: S1 File — The following are plotted (KOL always in red). Figures A, D, G, J, M, P, S, V, Y: KOL, HST(D) and AREA. Figures B, E, H, K, N, Q, T, W, Z: KOL, OACA, OACI, BVLA and BVLI. Figures C, F, I, L, O, R, U, X, Ø: KOL, DISP, OACC and BVLC. (GZ) [file pone.0119306.s001.gz › AllFigs/Figure_P.tif]

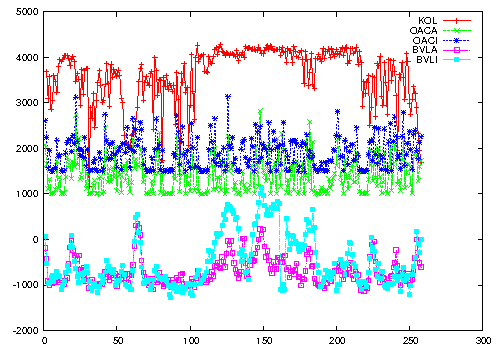

Supplement: S1 File — The following are plotted (KOL always in red). Figures A, D, G, J, M, P, S, V, Y: KOL, HST(D) and AREA. Figures B, E, H, K, N, Q, T, W, Z: KOL, OACA, OACI, BVLA and BVLI. Figures C, F, I, L, O, R, U, X, Ø: KOL, DISP, OACC and BVLC. (GZ) [file pone.0119306.s001.gz › AllFigs/Figure_Q.tif]

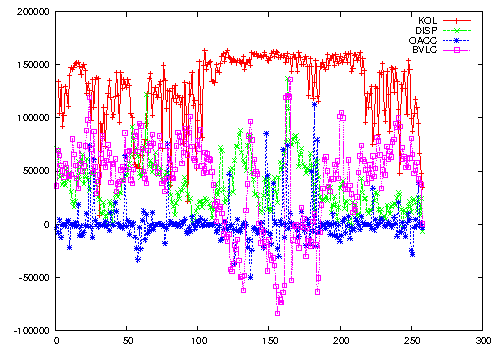

Supplement: S1 File — The following are plotted (KOL always in red). Figures A, D, G, J, M, P, S, V, Y: KOL, HST(D) and AREA. Figures B, E, H, K, N, Q, T, W, Z: KOL, OACA, OACI, BVLA and BVLI. Figures C, F, I, L, O, R, U, X, Ø: KOL, DISP, OACC and BVLC. (GZ) [file pone.0119306.s001.gz › AllFigs/Figure_R.tif]

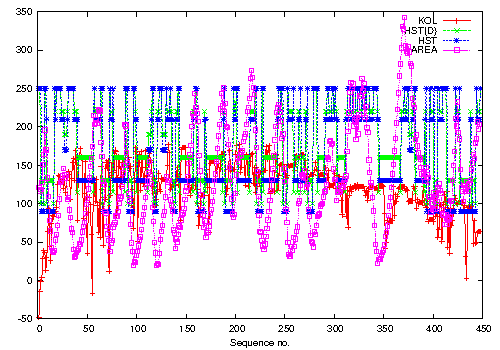

Supplement: S1 File — The following are plotted (KOL always in red). Figures A, D, G, J, M, P, S, V, Y: KOL, HST(D) and AREA. Figures B, E, H, K, N, Q, T, W, Z: KOL, OACA, OACI, BVLA and BVLI. Figures C, F, I, L, O, R, U, X, Ø: KOL, DISP, OACC and BVLC. (GZ) [file pone.0119306.s001.gz › AllFigs/Figure_S.tif]

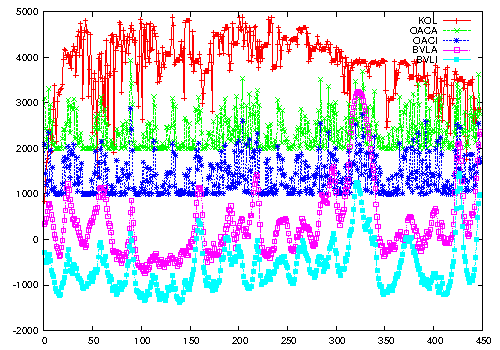

Supplement: S1 File — The following are plotted (KOL always in red). Figures A, D, G, J, M, P, S, V, Y: KOL, HST(D) and AREA. Figures B, E, H, K, N, Q, T, W, Z: KOL, OACA, OACI, BVLA and BVLI. Figures C, F, I, L, O, R, U, X, Ø: KOL, DISP, OACC and BVLC. (GZ) [file pone.0119306.s001.gz › AllFigs/Figure_T.tif]

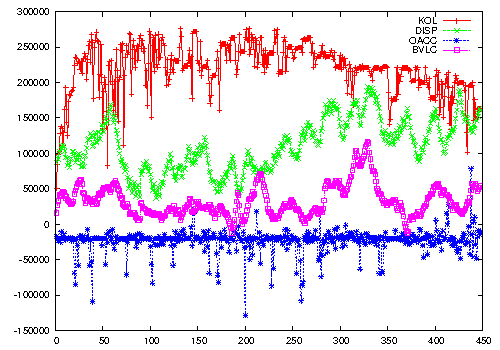

Supplement: S1 File — The following are plotted (KOL always in red). Figures A, D, G, J, M, P, S, V, Y: KOL, HST(D) and AREA. Figures B, E, H, K, N, Q, T, W, Z: KOL, OACA, OACI, BVLA and BVLI. Figures C, F, I, L, O, R, U, X, Ø: KOL, DISP, OACC and BVLC. (GZ) [file pone.0119306.s001.gz › AllFigs/Figure_U.tif]

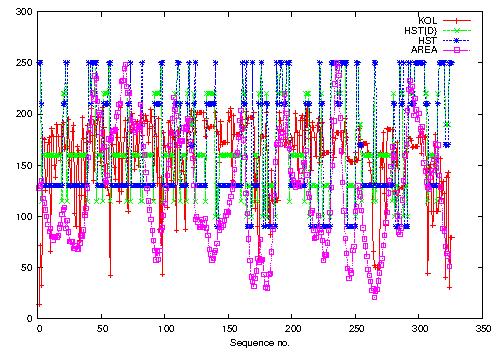

Supplement: S1 File — The following are plotted (KOL always in red). Figures A, D, G, J, M, P, S, V, Y: KOL, HST(D) and AREA. Figures B, E, H, K, N, Q, T, W, Z: KOL, OACA, OACI, BVLA and BVLI. Figures C, F, I, L, O, R, U, X, Ø: KOL, DISP, OACC and BVLC. (GZ) [file pone.0119306.s001.gz › AllFigs/Figure_V.tif]

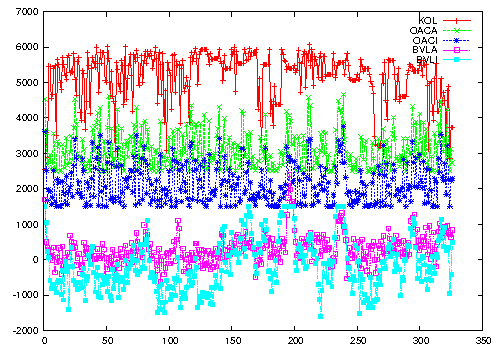

Supplement: S1 File — The following are plotted (KOL always in red). Figures A, D, G, J, M, P, S, V, Y: KOL, HST(D) and AREA. Figures B, E, H, K, N, Q, T, W, Z: KOL, OACA, OACI, BVLA and BVLI. Figures C, F, I, L, O, R, U, X, Ø: KOL, DISP, OACC and BVLC. (GZ) [file pone.0119306.s001.gz › AllFigs/Figure_W.tif]

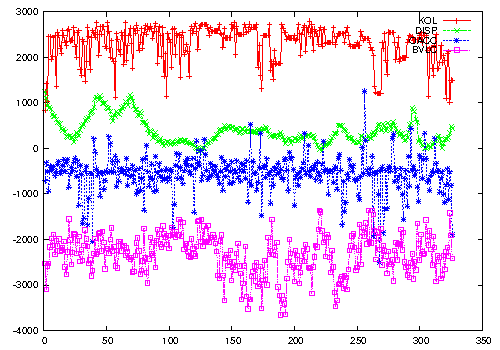

Supplement: S1 File — The following are plotted (KOL always in red). Figures A, D, G, J, M, P, S, V, Y: KOL, HST(D) and AREA. Figures B, E, H, K, N, Q, T, W, Z: KOL, OACA, OACI, BVLA and BVLI. Figures C, F, I, L, O, R, U, X, Ø: KOL, DISP, OACC and BVLC. (GZ) [file pone.0119306.s001.gz › AllFigs/Figure_X.tif]

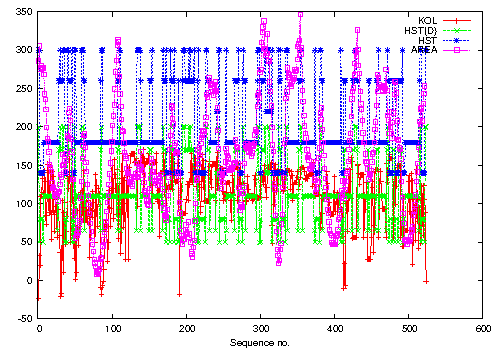

Supplement: S1 File — The following are plotted (KOL always in red). Figures A, D, G, J, M, P, S, V, Y: KOL, HST(D) and AREA. Figures B, E, H, K, N, Q, T, W, Z: KOL, OACA, OACI, BVLA and BVLI. Figures C, F, I, L, O, R, U, X, Ø: KOL, DISP, OACC and BVLC. (GZ) [file pone.0119306.s001.gz › AllFigs/Figure_Y.tif]

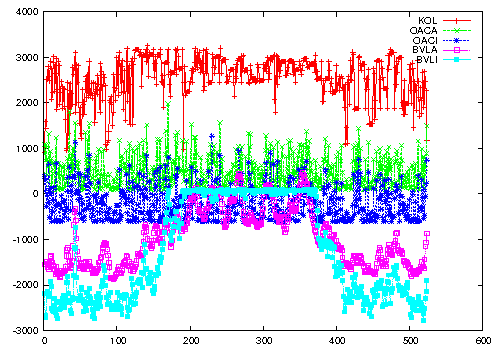

Supplement: S1 File — The following are plotted (KOL always in red). Figures A, D, G, J, M, P, S, V, Y: KOL, HST(D) and AREA. Figures B, E, H, K, N, Q, T, W, Z: KOL, OACA, OACI, BVLA and BVLI. Figures C, F, I, L, O, R, U, X, Ø: KOL, DISP, OACC and BVLC. (GZ) [file pone.0119306.s001.gz › AllFigs/Figure_Z.tif]
