# Supplementary material for: Prediction of Protein Structural Features from Sequence Data Based on Shannon Entropy and Kolmogorov Complexity
Source: PLoS One. 2015 Apr 9;10(4):e0119306. doi: 10.1371/journal.pone.0119306 (PMC4391790; doi:10.1371/journal.pone.0119306)
Supplement: S1 File — The following are plotted (KOL always in red). Figures A, D, G, J, M, P, S, V, Y: KOL, HST(D) and AREA. Figures B, E, H, K, N, Q, T, W, Z: KOL, OACA, OACI, BVLA and BVLI. Figures C, F, I, L, O, R, U, X, Ø: KOL, DISP, OACC and BVLC. (GZ) [file pone.0119306.s001.gz › AllFigs/Appendix.doc]

**Appendix**

**1) *Explanatory note regarding Kolmogorov complexity.***

Ever since the 1940's most methods of quantifying information content have been based in one way or another on the notion of Shannon entropy. This is widely used in telecommunications, informatics and computer science, and is a most efficient way of measuring (the fidelity of) information transfer but it says nothing about the quality of the information itself, only the quantity. Other mathematicians wanted to address the "quality" issue (even “aesthetic quality”), most notably Kolmogorov [39]. This laudable aim was never fully accomplished by him (or anyone else to date) but an essential first step was accomplished. Kolmogorov invented a new measure of information content, based on (this is the simplest explanation) the notion of "how far one can compress a message without losing any information". For example the prime number 104543 cannot be written in any simplified form while a number close to it, 102400 is the square of 320, and 320 can be written as 5*2*2*2*2*2*2 or 5*2^6. The latter number has a much lower Kolmogorov complexity. This way of reckoning complexity is increasingly coming into use in physics, chemistry, econometrics and bioinformatics, number theory, cryptography and of course computer science.

It needs to be pointed out that when measuring the compressibility of a string of symbols, the ordering in which the members of the string can be important. In the above example the operation is “commutative under the operation of multiplication” to use strict mathematical terminology. In terms that the general reader can understand: the order in which you take the symbols for your operation doesn't matter. In literature, it certainly will make a difference. Likewise, in the science of cryptography, the order matters and it is one of the reasons why cryptography exists. In bioinformatics, as here: it can make a difference in the sense that, say, at one residue position the sequence AAAAAST looks and indeed is more “compressible” than AASAATA or SAAATAA. But if you do that same reordering at another site you reorder e.g. GPGGPGG to GPGGPGG or GPGGGGP or GPGGGPG or GPGGGGP etc, which will make a noticeable difference but might cancel the “ordering effect” at the previous site. But all these cases deal with short “words”. One of the most important directives in bioinformatics however is that it is not enough to compare 7 aligned sequences as here, it has to be at least of the order of many hundreds. When that is done, an alignment like AAAAAST gets buried in something much bigger and then this kind of “ordering” begins to lose its significance. The author has conducted trials that address this issue and the results show that there is no significant “ordering effect” once NALIGN – the number of sequences in the alignment is more than a hundred or so (data available from the author).

**2) *Explanatory note regarding calculation of areas as in AREA.***

The algorithm used for determining areas of triangles formed by two CA atoms in the protein chain together with the global center of gravity of the protein is based on the classical Heron's formula, which is best expressed as a Cayley-Menger determinant wherein A is the desired area and the d values are the lengths of the sides that form the triangle where each apex is numbered 1,2,3:

| 1 1 1 1 |

| 1 0 d_12_^2^ d_13_^2^ |

A**2 =	- 1/16	|	1	d_12_^2^	0	d_23_^2^	|

| 1 d_13_^2^ d_23_^2^ 0 |
